# Postnatal Leptin Promotes Organ Maturation and Development in IUGR Piglets

**DOI:** 10.1371/journal.pone.0064616

**Published:** 2013-05-31

**Authors:** Linda Attig, Daphné Brisard, Thibaut Larcher, Michal Mickiewicz, Paul Guilloteau, Samir Boukthir, Claude-Narcisse Niamba, Arieh Gertler, Jean Djiane, Danielle Monniaux, Latifa Abdennebi-Najar

**Affiliations:** 1 UPSP EGEAL Institut Polytechnique LaSalle Beauvais, Beauvais, France; 2 INRA, UMR85 Physiologie de la Reproduction et des Comportements, Nouzilly, France; 3 CNRS, UMR7247, Nouzilly, France; 4 Université François Rabelais de Tours, Tours, France; 5 IFCE, Nouzilly, France; 6 INRA UMR 703 Ecole Nationale Vétérinaire, Nantes, France; 7 INRA, Unité 1341, Nutrition et Adaptations Digestives, Nerveuses et Comportementales (ADNC), Saint Gilles, France; 8 Tunis El Manar University, Faculty of Medicine of Tunis, Children’s Hospital, Department of Pediatrics “C”, RU12SP09, Tunis, Tunisia; 9 The Robert Smith Faculty of Agriculture, Food and Environment, Rehovot, Israel; 10 INRA, Centre de recherche, Jouy-en-Josas, France; The Ohio State University, United States of America

## Abstract

Babies with intra-uterine growth restriction (IUGR) are at increased risk for experiencing negative neonatal outcomes due to their general developmental delay. The present study aimed to investigate the effects of a short postnatal leptin supply on the growth, structure, and functionality of several organs at weaning. IUGR piglets were injected from day 0 to day 5 with either 0.5 mg/kg/d leptin (IUGR*Lep*) or saline (IUGR*Sal*) and euthanized at day 21. Their organs were collected, weighed, and sampled for histological, biochemical, and immunohistochemical analyses. Leptin induced an increase in body weight and the relative weights of the liver, spleen, pancreas, kidneys, and small intestine without any changes in triglycerides, glucose and cholesterol levels. Notable structural and functional changes occurred in the ovaries, pancreas, and secondary lymphoid organs. The ovaries of IUGR*Lep* piglets contained less oogonia but more oocytes enclosed in primordial and growing follicles than the ovaries of IUGR*Sal* piglets, and FOXO3A staining grade was higher in the germ cells of IUGR*Lep* piglets. Within the exocrine parenchyma of the pancreas, IUGR*Lep* piglets presented a high rate of apoptotic cells associated with a higher trypsin activity. In the spleen and the Peyer’s patches, B lymphocyte follicles were much larger in IUGR*Lep* piglets than in IUGR*Sal* piglets. Moreover, IUGR*Lep* piglets showed numerous CD79^+^cells in well-differentiated follicle structures, suggesting a more mature immune system. This study highlights a new role for leptin in general developmental processes and may provide new insight into IUGR pathology.

## Introduction

Human neonates born with intra-uterine growth restriction (IUGR) often experience adverse perinatal outcomes and present an increased risk of mortality [1]. Their general developmental delay affects the growth and functional properties of various organs, leading to immediate defects in key biological functions such as immunity and digestion [2], [3]. This situation renders difficult their adaptation to extra-uterine life and contributes to the development of diseases, impacting their survival and increasing the intensity of their hospital care and the associated costs [1]. Reduced fetal growth also exerts adverse effects on reproductive functions; in females, IUGR is associated with the development of reproductive disorders in later life, including altered timing of onset of puberty, premature adrenarche, and earlier menopause with subsequent fertility problems [4], [5]. Later in life, IUGR babies with suboptimal growth are at increased risk of metabolic programming and cardiovascular disease [6].

The levels of numerous hormones such as cortisol, growth hormone, insulin-like growth factors, and thyroid hormones are altered in IUGR fetuses and neonates [7], [8], [9]. These hormones are all key regulators of growth and organ maturation, and together they orchestrate the harmonious development of the individual. Therefore, the perturbation of their secretion or action during fetal and perinatal life in IUGR induces persistent changes in organ structure and function, with immediate and long-lasting detrimental effects. Leptin has been added to the list of endocrine factors that are altered following growth restriction during fetal and early postnatal life in human and rodents. Leptin levels are low in human IUGR neonates, which may contribute to the long-term programming of metabolic syndrome [10], [11]. Leptin is a cytokine produced mainly by the adipose tissue; it has been extensively studied for its key role in the central regulation of food intake and energy expenditure [12]. Strategies of leptin supplementation in early postnatal life have been beneficial for the correction of the short- and long-term IUGR phenotypes in terms of food intake regulation, body weight (BW) gain, and body composition in rats and pigs particularly in females [13], [14].

In addition to its contribution to the regulation of energy balance, leptin’s pleiotropic effects are involved in the regulation of a wide range of biological functions, notably reproduction, osteogenesis, hematopoesis, and immunity [15]. A new role for leptin in developmental processes has recently emerged from several studies, mostly in rodents. In newborn rats, a dramatic increase in leptin levels occurs during the first two weeks of life independent of fat accretion and BW [16], [17]. During this period, leptin acts as a neurotrophic factor to coordinate the establishment of the hypothalamic neuronal network responsible for food intake regulation [18]. For organs other than the brain, only a few studies have investigated the developmental effects exerted by leptin, but its stimulation of proliferation and differentiation of various cell types is well documented [15]. Our recent work in rodents clearly demonstrated that leptin may constitute a key hormone for the postnatal maturation of numerous peripheral organs involved not only in metabolic functions but also in immunity and reproduction [19]. However, in rodents the temporal windows of development for many organs differ from those of humans, making it difficult to directly extrapolate these results to humans. Pigs are an advantageous model for many human physiological aspects and for the timing of development and maturation of many human organs. Interestingly, in pigs, IUGR occurs naturally is frequently due to the hyper prolificacy of sows in breeding, and IUGR results in similar long-term pathological consequences as in humans, including increased adiposity, hypertension, cardiovascular risk, and glucose intolerance [20], [21], [22].

While leptin supplementation to piglet neonate had no effects on general growth [23], we previously showed that treatment of IUGR female piglets during the first 10 days of life enhanced their ponderal index and linear growth and was associated with an apparent improvement in the growth of several organs [13]. To the best of our knowledge, there are no published reports of the mechanisms of action of leptin on organ growth and maturation in IUGR. The primary purpose of this study was to elucidate some of the physiological processes that occur at the cellular level of the reproductive, immune and gastrointestinal, systems after leptin neonatal supply. We intended to shorten the period of treatment to 5 days in order to check the beneficial effects for an applicative clinical use.

## Materials and Methods

### Ethic Statement

The experimental protocol was designed in compliance with recommendations of the French law (Decret: 2001-464 29/05/01) and EEC (86/609/CEE) for the care and use of laboratory animals under the certificate of authorization to experiment on living animals. All procedures were approved by the Animal Ethics Committee “Comité Régional d’Ethique sur l’Expérimentation Animale, Ile-de-France Sud”.

### Animals and Experimental Design

We used commercial crossbred pigs (1/4 Large White×1/4 Duroc×1/4 Pietrain×1/4 in the Landrace) from our experimental farm (LaSalle Beauvais, France). Among the non-gilt in the herd, 10 sows were chosen when they produced at least 13 piglets at delivery. Piglets weighing the average birth BW of the herd (1.476±0.043 Kg) were identified as normal BW. Within 24 h after birth (day 0; d0), 40 female piglets (0.975±0.26 Kg) from the 10 litters were selected on the basis of their BW. These 40 piglets were considered as IUGR piglets since their mean BW was around 1.000 kg and was 34% lower than that of the normal BW piglets. Piglets were cross-fostered to minimize competition between piglets and equalize litter sizes. The piglets remained with the sow and had access to normal suckling until sacrifice at d21. At d7, the piglets were allowed free access to a starter commercial diet complementing maternal milk feeding in accordance with the European recommendations for animal welfare in breeding (2001/91/CE, November 9, 2001). Measures of BW were taken daily until d7 and again before euthanasia (d21).

### *In vivo* Leptin Treatment

Recombinant porcine leptin was prepared as reported previously. The protein was 98% pure by SDS-PAGE and over 95% monomeric. It was fully active in an *in vitro* bioassay in Baf/3 cells stably transfected with the long form of human leptin receptor [24]. Its endotoxin content was <0.02 ng/µg protein. In a preliminary experiment, we determined the optimal dose of porcine leptin administration to obtain the longest duration of high leptin levels. We found that administration of 0.5 mg/kg leptin leads to circulating leptin concentrations of up to 40 ng/ml, 1 h after injection, which persist for at least 6 h. Leptin levels assessed in blood samples collected one hour after injection were >10 fold higher in IUGR*Lep* piglets than physiological levels measured in IUGR*Sal* animals [13]. In the present experiment, IUGR animals received therefore daily intramuscular injections of either saline (IUGR*Sal*, n = 20) or 0.5 mg/kg porcine recombinant leptin (IUGR*Lep*, n = 20) from d0 to d5.

### Determination of Plasma Hormones and Metabolites Levels

Blood samples were collected at sacrifice (d21) in heparinized tubes, by venopuncture of the subclavian vein. After centrifugation at 2,300×g for 10 min at 4°C, plasma was stored at −20°C until analysis. Glucose, cholesterol and triglycerides concentrations were determined using commercial kits based on enzymatic technique coupled with colorimetric detection (Glucose PAP Kit, Cholesterol Kit, Triglycerides MONO SL NEW Kit, ELITech, France Biotechnologies).

### Tissue Collection and Sampling

At d21, animals were euthanized by intraperitoneal injection of 90 mg/kg sodium thiopental (Nesdonal, Rhône-Mérieux, France). The liver, stomach, heart, lungs, pancreas, kidneys, and spleen were dissected and weighed. The small intestine was separated from the mesentery, flushed with cold saline, blotted dry with absorbing paper, weighed, and a fragment of jejunum was collected. Samples of heart, pancreas, kidney, liver, spleen, and jejunum were fixed with 4% neutral buffered formalin and embedded in paraffin wax for histological analysis. Samples of a part of the small intestine (10 cm from the mid-jejunum) were frozen in liquid nitrogen and stored at −80°C for biochemical studies. After dissection, one ovary of each piglet was fixed in aqueous Bouin and embedded in paraffin for further histology and immunohistochemistry. The second ovary was frozen in liquid nitrogen and stored at −80°C for western blot analysis.

### Digestive Enzyme Activities

Pancreatic and intestinal tissues were scraped with a glass slide, weighed, and homogenized in cold distilled water (1 g of tissue/5 mL of distilled water). After centrifugation for 5 min at 1,000×g at 4°C, the protein contents were determined by the Lowry method. In pancreatic tissue, the activities of trypsin and α-amylase were assayed as previously described [25], [26]. In intestinal mucosa, the activity of aminopeptidase N was measured with L-leucyl-*p*-nitroanilide as substrate [27]. Enzyme activities were calculated as µmol hydrolyzed substrate per min (IU) and expressed as specific (IU per g protein) or total (IU/kg piglet BW) activity.

### Histological Analysis

Representative panel of organs and tissues (heart, spleen, liver, pancreas, kidney, jejunum) embedded in paraffin wax were transversally cut into 5 µm-thick sections, fixed to positive charge slides, and stained using a routine hematoxylin-eosin-safranine staining method. All samples were evaluated by a skilled pathologist in a double-blind manner, and lesions were systematically recorded. Afterward, complementary quantitative analyses were carried out on kidney, pancreas, jejunum, and Peyer’s patches. Image analysis was performed using a digital camera (Nikon DXM 1200, Champigny, France) combined with image-analysis software (Nikon Imaging Software). For each histological parameter, preliminary intra-observer agreement was tested by reproducing the measure three times with the same sample to determine the coefficient of reproducibility (CR).

In kidney samples, as many microscopic fields as necessary to observe at least 100 glomeruli per sample were randomly selected in the cortical portion of the sample (23±5 glomeruli per field). Glomeruli were numbered, with a CR of 100%, and glomerular density was calculated in glomeruli/µm^2^. Glomerular mean size was determined using the Ferret minimal diameter of at least 70 glomeruli per sample (CR of 95.3%).

In pancreatic samples, the number of exocrine cells, apoptotic events, and mitotic events were numbered in 10 randomly selected high-magnification fields. Additionally, the numbers of acinar cells were determined, and the area of pancreatic acini was measured using the Ferret minimal diameter.

In jejunal segments of the intestine, six intermediate-powered fields were randomly selected. Villous height and crypt depth were measured (CR of 94.7%), and mucosal thickness was estimated for each sample as the sum of the villous height and crypt depth measured in at least 10 different locations (CR of 96.1%).

In Peyer’s patches located along the jejunum wall, the surface areas of the whole Peyer’s patches and the follicular area were determined in 10 Peyer’s patches per sample (CR of 98.8%). For each Peyer’s patch, the ratio between the follicle area normalized to the total surface of the Peyer’s patches was calculated, and an average of the ratios obtained for the 10 Peyer’s patches was then calculated.

Seven-micron sections of ovaries were taken, fixed onto glass slides, and stained with hematoxylin. Germ cell counting was performed on five ovarian sections per animal. On each section, four different microscopic fields were analyzed with a 40× objective, corresponding to a total tissue area of 1.24 mm^2^ analyzed per animal. Six categories of germ cells were identified and counted: oogonia, oocytes in meiotic prophase, oocytes in the dictyate stage grouped within germ cell cysts, and oocytes included in primordial, primary, and secondary follicles. We also examined degenerated cells that presented either pyknosis or nuclear fragmentation and abnormal cytoplasm. A total of 3318 and 2609 healthy germ cells were analyzed in IUGR*Sal* and IUGR*Lep* animals, respectively. Data were expressed as percentages of the total numbers of healthy germ cells in each group.

### Immunohistochemistry

Spleen sections were obtained from paraffin-embedded samples. Dewaxing was performed using serial baths in methylcyclohexane and in ethanol solutions of decreasing concentration (100%, 95%, 80% by volume in water). After washing in distilled water, antigen retrieval was performed in boiling 10 mM citrate buffer (pH 6.0) for 40 min. Endogenous peroxidase activity was inhibited by incubating the samples in 3% H_2_O_2_ in water for 10 min. The samples were blocked for 20 min in a solution containing 10% normal goat serum and 2% PBS. The samples were incubated with a CD79 antibody (clone HM57, Dako, Glostrup, Denmark) diluted 1∶50 in 2% BSA for 1 h at 37°C. Unbound primary antibody was removed by washing the samples in PBS. Signal amplification and recognition of the primary antibody were performed by incubating the samples with a biotinylated rabbit anti-mouse Ig secondary antibody (Dako) diluted 1∶300 in PBS/2%BSA. The samples were washed in PBS to remove unbound secondary antibody and incubated for 30 min at room temperature with the streptavidin-horseradish peroxidase complex (streptavidin-HRP, Dako) diluted 1∶300 in PBS. After three washes in PBS, the signal was visualized with 3,3′-diaminobenzidine tetrahydrochloride (DAB, Dako).

The presence of leptin receptors was investigated by immunohistochemistry in ovaries. After dewaxing and rehydrating the sections, boiling citrate buffer (Vector Laboratories, Burlingame, CA, USA) was used to retrieve the antigen sites. Endogenous peroxidases were quenched with 0.3% H_2_O_2_. After washing, the sections were incubated with 1∶250 rabbit anti-mouse leptin receptor antibody (Ob-R (M-18)-R, sc-1834-R; Santa Cruz Biotechnology Inc, Heidelberg, Germany) in a humidified chamber overnight at 4°C. After rinsing, the sections were incubated with biotinylated horse anti-mouse/rabbit IgG (Vector Laboratories, final dilution 1∶800) for 4 h at room temperature, then with the avidin-biotin HRP complex (Vectastain Elite ABC Kit, Vector Laboratories) for 30 min. Staining was carried out after the addition of DAB in the presence of 0.0072% H_2_O_2_ for 10 min, and the sections were counterstained with hematoxylin for 5 min. In order to verify antibody specificity, sections were co-incubated with the peptide used as the immunogen for Ob-R antibody preparation (Ob-R (M-18) P, sc-1834 P; Santa Cruz Biotechnology Inc) at 10 µg/ml and 1∶250 Ob-R antibody.

To further assess changes in ovarian follicular growth activation after leptin treatment, the expression of FOXO3A, a suppressor of follicle growth activation, was studied via immunohistochemistry on ovary sections from the IUGR*Sal* and IUGR*Lep* animals using a rabbit anti-human FOXO3A antibody (# 9467, Cell Signaling Technology, Danvers, MA, USA) at a dilution of 1∶250 as described above. The number of germ cells with diffuse cytoplasmic staining was evaluated semi-quantitatively in two zones of ovary sections (the sub-epithelial zone and the deep ovarian cortex), each corresponding to a tissue area of 0.62 mm^2^ analyzed per animal. Four grades of staining were defined, corresponding to the number of stained germ cells per microscopic field:<5 (grade 0), 5–25 (grade 1), 25–50 (grade 2), and >50 (grade 3). In each zone, the number of germ cells with a strong perinuclear staining was also counted.

### Protein Extraction and Western Blot Analysis

Lysis buffer (1 ml) was added to a half ovary. Spermatozoa which were used as a positive control for Ob-R expression [28] were washed in PBS twice, then pelleted by centrifugation (10 min, 15 000 g at room temperature) before lysis. After crushing and sonication of the samples, protein extracts were recovered by centrifugation (30 min, 11,000×g at 4°C). Before loading, concentrated reducing Laemmli buffer containing 80 mM DTT at final concentration was added to all protein extracts and samples were boiled for 5 min. Samples were pooled according to their treatment, thereby 3 pools were obtained for IUGR*Lep* and 2 pools for IUGR*Sal*. Protein extracts were resolved on 10% SDS-PAGE gels and transferred on nitrocellulose membranes. Antibodies were added overnight at 4°C, at dilution of 1∶200 for FOXO3a and 1∶260 for leptin receptor. After several washes, immunoreactivity was detected through specific HRP-conjugated secondary antibodies at a dilution of 1∶10,000. The enhanced chemiluminescence ECL Plus kit (Amersham Biosciences, Orsay, France) was used for revelation according to the manufacturer’s instructions.

### Statistical Analysis

The data were analyzed by using GraphPad Prism 2.0 Software and were evaluated with the nonparametric Mann-Whitney test. The percentages of germ cell types present in the ovaries of IUGR*Sal* and IUGR*Lep* piglets were analyzed using Pearson’s chi-squared test. For all analyses, the level of significance was set at p<0.05. All values are expressed as mean ±standard error of the mean (SEM).

## Results

### Effect of Leptin Treatment on Postnatal Growth and Organ Development

**Figure 1:**
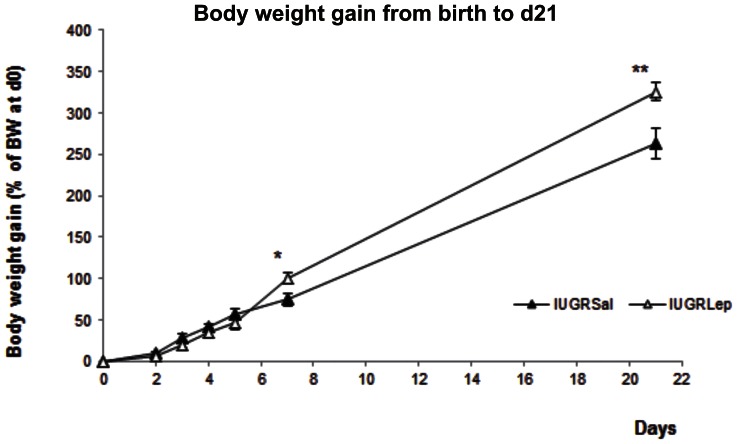
Growth and organ development of IUGR piglets treated with either saline (IUGR*Sal*) or leptin (IUGRL*ep*). (A) Morphometric parameters and organ weights at weaning (d21). (B) Evolution of body weight gain from birth to d21. BW: Body weight. Values represent the mean ± SEM, (n = 20 per group), *: p<0.05; **: p<0.01, for leptin effect in IUGR piglets.

At d21, the BW of the IUGR*Sal* piglets tended to be lower than those of the IUGR*Lep* piglets (IUGR*Sal*: 3.79±0.22 kg, IUGR*Lep*: 4.19±0.17 kg), with a difference of 10.6% between the two groups (Table 1). In terms of linear growth, no significant difference was observed between the two groups (Table 1). From birth to d5 of the follow-up period, the growth rates were similar in the leptin and saline groups (Figure 1; IUGR*Sal*: 56.81±8.25%, IUGR*Lep*: 46.93±6.56%). At d7, while the IUGR*Sal* piglets continued to follow the same growth trajectory, there was an acceleration of weight gain in the leptin-treated animals that resulted at d21 in a higher final percentage of BW gain (IUGR*Sal*: 262.6±11.1%, IUGR*Lep*: 325.9±18.8%, p<0.01).

**Table 1:** Morphometric parameters and organ weights at weaning (d21) of IUGR piglets treated with either saline (IUGR*Sal*) or leptin (IUGR*Lep*).

Parameters	IUGR*Sal*	IUGR*Lep*
***Morphometric parameters***		
Body weight (BW) (kg)	3.79 ±0.22	4.19±0.17
Length (cm)	67.5±0.5	64.5±1.8
***Organ relative weights (% of BW)***		
Liver	2.57±0.14	3.25**±0.15
Lungs	1.45±0.07	1.52±0.03
Heart	0.71±0.06	0.70±0.03
Spleen	0.23±0.01	0.27*±0.01
Small intestine	3.59±0.10	4.44*±0.36
Kidney	0.35±0.01	0.42*±0.02
Pancreas	0.13±0.01	0.17*±0.01

Values represent the mean ± SEM, (n = 20 per group),

* p<0.05;

** p<0.01, for leptin effect in IUGR piglets.

No significant differences between the IUGR*Lep* and IUGR*Sal* groups were detected for the relative weights of the collected organs, except for the liver (p<0.01), spleen, kidney, small intestine and pancreas (p<0.05), which were significantly heavier in the leptin-treated group (Table 1).

### Effect of Leptin on Metabolic Parameters

Leptin supplementation did not affect the level of triglycerides, glucose and cholesterol at day 21 (triglycerides: IUGR*sal*: 2,15 mmol/L ±0,33 mmol/L n = 10; IUGR*lep*: 1,58 mmol/L ±0,12 mmol/L n = 10) (glucose: IUGR*sal*: 9.39 mmol/L ±1.14 mmol/L, n = 4; IUGR*lep: 8.86 *mmol/L ±1.18 mmol/L, n = 4), (cholesterol: IUGR*sal* : 2.15 mmol/L ±0.33 mmol/L n = 10; IUGR*lep:* 1.58 mmol/L±0,12 mmol/L, n = 10).

### Effect of Leptin on Organ Histological Structure and Functionality

For the ovaries and the organs whose relative weights appeared to be highly modified by leptin treatment, we performed further analysis with histomorphometrical and immunohistochemical approaches. These analyses were also conducted for the gastrointestinal tract (liver, small intestine, and pancreas), the kidneys, and secondary lymphoid organs (spleen and Peyer’s patches). Western blot analysis was additionally performed on ovarian samples to study the expression of leptin receptor and FOXO3A.

**Figure 2:**
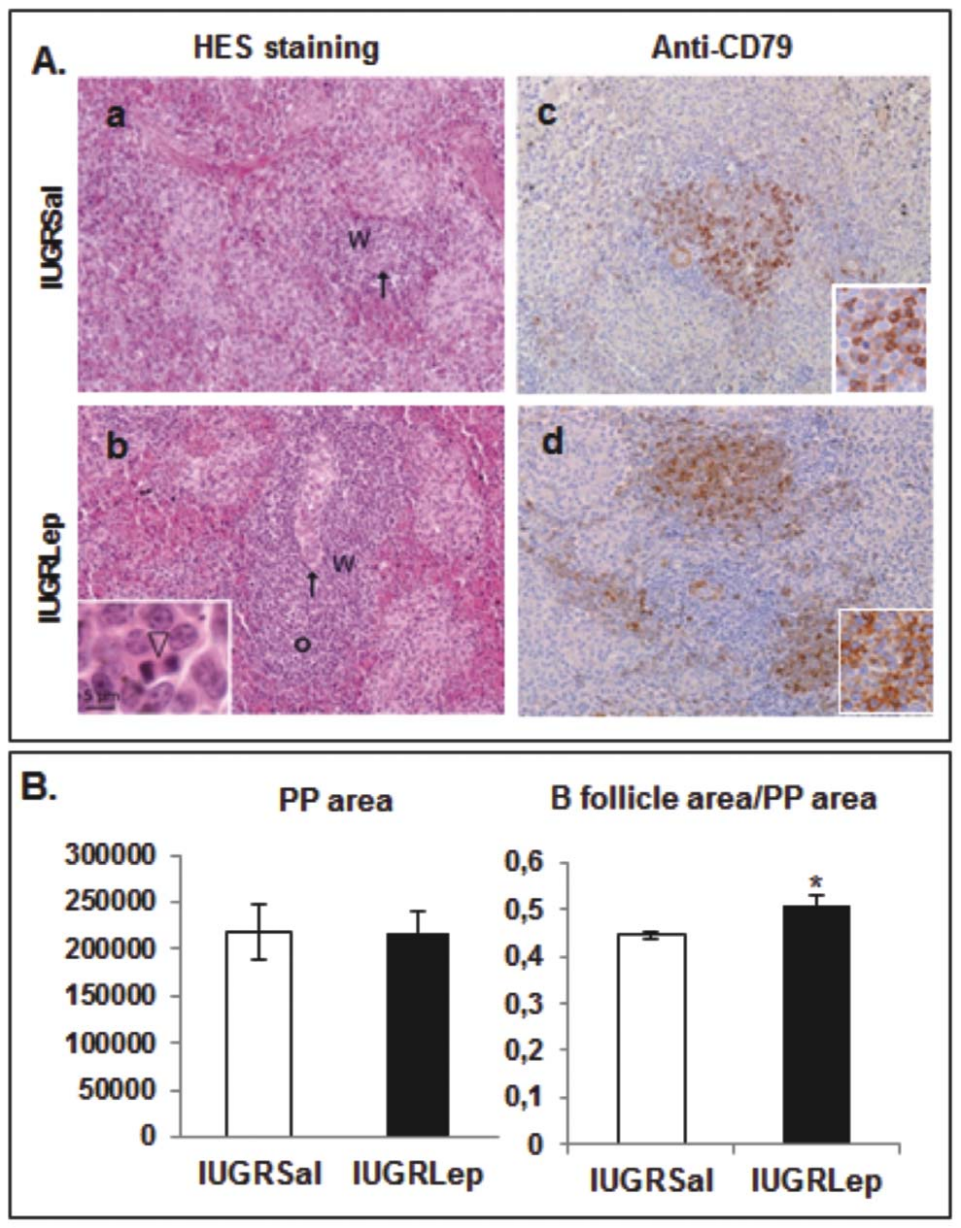
Ovarian histological structure and expression of leptin receptors in ovaries of IUGR piglet treated with either saline (IUGR*Sal*) or leptin (IUGR*Lep*). (A) Germ cell populations in piglet ovaries at d21. a: oogonia (oog) within germ cell nests or cysts, b: oocytes in meiotic prophase (proph) within germ cell nests or cysts, c: oocytes in dictyate stage (cysts) grouped within germ cell cysts, d: oocytes enclosed in primordial follicles (foll prim), e: oocytes enclosed in primary follicles (foll I), f: oocyte enclosed in a secondary follicle. (foll II). The arrows indicate examples of germ cells. Bar = 20 µm, g: Percentages of different germ cell types in IUGR*Sal* and IUGR*Lep* piglet ovaries. (B) Immunostaining for leptin receptors in piglet ovaries at d21. Immunostaining of piglet ovarian sections using Ob-R antibody, without (a) and with (b)displacement by the peptide used as immunogen for Ob-R antibody preparation; o = oocyte. Bar = 20 µm. (C) Detection of leptin receptors by Western Blot using Ob-R antibody in piglet IUGR*Sal* and IUGR*Lep* ovaries at d21 and in a spermatozoa lysate as a positive control. Six Ob-R isoforms were observed, including the long form (Ob-Rb at 120 kDa) and the first short form (Ob-Ra at 90 kDa).

Various types of germ cells were identified in piglet ovaries (Figure 2 A a–f). Both IUGR*Sal* and IUGR*Lep* ovaries contained oogonia, oocytes in meiotic prophase, oocytes in the dictyate stage grouped within germ cell cysts, and oocytes in primordial, primary, and secondary follicles. The proportions of the germ cell types differed between IUGR*Sal* and IUGR*Lep* piglet ovaries (Figure 2A g). Leptin-treated piglets had lower percentages of oogonia (p<0.001) and oocytes in meiotic prophase (p<0.001), but higher percentages of oocytes in the dictyate stage within germ cell cysts (p<0.05) and oocytes within primordial (p<0.001) and primary (p<0.001) follicles.

Leptin receptors were detected in oogonia and oocytes in the ovaries of all piglets (Figure 2B). Western blotting experiment highlighted the presence of six isoforms of leptin receptor in the piglet IUGR*Sal* and IUGR*Lep* ovaries (Figure 2C). The long form of the receptor (Ob-Rb) is at 120 kDa. The second longer form of the leptin receptors (90 kDa band) might correspond to the Ob-Ra isoform, according to data available for the different isoforms of the human receptor (accession number P48357, UniProtKB). The relative expression of the different isoforms seems to be tissue specific, as illustrated by the high expression of the 60 kDa form in the spermatozoa.

**Figure 3:**
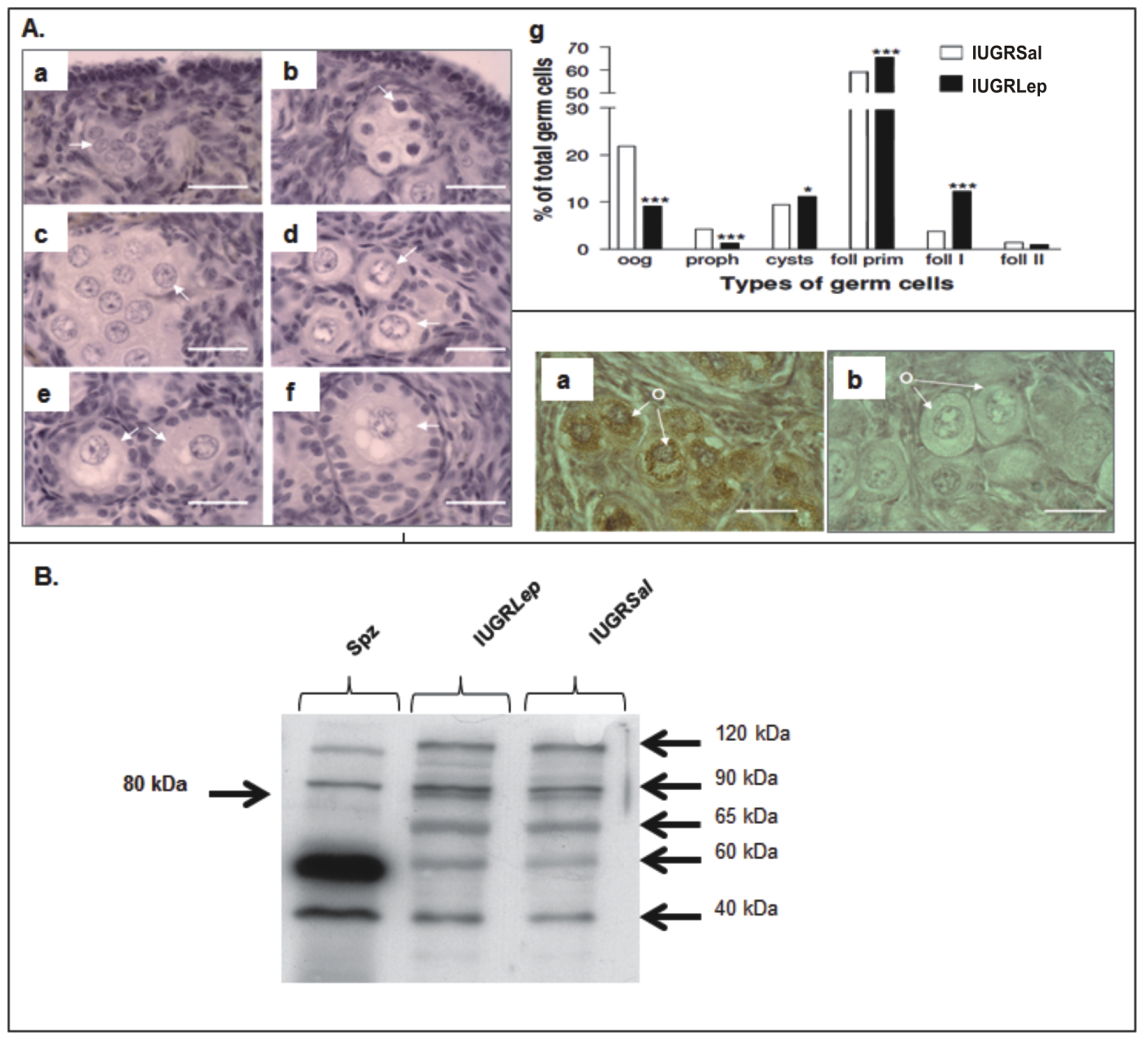
FOXO3A expression in ovaries of IUGR piglet treated with either saline (IUGR*Sal*) or leptin (IUGR*Lep*). (A) Detection of FOXO3A by Western Blot using FOXO3A antibody in piglet IUGR*Sal* and IUGR*Lep* ovaries at d21. (B) Immunostaining of piglet ovarian sections using FOXO3A antibody (a, b and c) showing a diffuse cytoplasmic staining in the oocytes of primary follicles (a), a strong perinuclear (arrows) and diffuse cytoplasmic staining in the oocytes of primordial follicles (b) and a strong perinuclear staining in the oocytes of secondary follicles (c), Bar = 20 µm. Graph in (d) shows FOXO3A staining grade in germ cells; four grades of staining were defined, corresponding to numbers of stained germ cells <5 (grade 0), 5–25 (grade 1), 25–50 (grade 2), and >50 (grade 3) per microscopic field (objective ×40). Graph in (e) shows the numbers of oocytes with a strong perinuclear staining. Data in panels d and e represent results of germ cell counting in the sub-epithelial and in the deep zones of ovarian cortex of IUGR*Sal* (n = 6) and IUGR*Lep* (n = 8) piglet ovaries, each zone corresponding to a tissue area of 0.62 mm2 analyzed per animal. Values represent the mean ± SEM, *: p<0.05; **: p<0.01, for leptin effect in IUGR piglet.

Western blotting for FOXO3A revealed the presence of a band corresponding to the expected molecular weight of 97 kDa in all piglet ovaries (Figure 3A). Detailed cellular analysis by immunohistochemistry of FOXO3A expression in IUGR*Sal* and IUGR*Lep* piglet ovaries showed diffuse cytoplasmic staining of FOXO3A in all germ cell types, and a strong perinuclear staining in some oocytes (Figure 3B a–c). The FOXO3A staining grade of the germ cells was higher (p<0.01) in the sub-epithelial and deep zones of the ovarian cortex (Figure 3B d) and the number of oocytes with strong perinuclear staining was also higher (p<0.05) in the sub-epithelial zone of the ovarian cortex (Figure 3B e) in leptin-treated piglets versus untreated piglets.

At d21, we did not observe any histological differences in the appearance of the jejunum in the two groups: Villi lengh (IUGR*Sal*: 374 µm ±77 µm, n = 6, R*Lep*: 345 µm ±29 µm, n = 6), crypt depth (IUGR*Sal*: 162 µm ±33 µm, n = 6; IUGR*Lep*: 169 µm ±21 µm, n = 6) and mucosa (IUGR*Sal*: 536 µm ±103 µm, n = 6; IUGR*Lep*: 514 µm ±46 µm, n = 6). Similarly, neither the total protein content (IUGR*Sal*: 4,912 mg/Kg BW ±1487 mg/Kg BW, n = 6; R*Lep*: 3,247 mg/Kg BW ±1036 mg/Kg BW, n = 6) nor the aminopeptidase N activity (IUGR*Sal*: 225,000 UI/Kg BW ±22,000 UI/Kg BW, n = 6; R*Lep*: 187,000 UI/Kg BW±65,000 UI/Kg BW, n = 6) of the jejunum significantly differed between groups.

**Figure 4:**
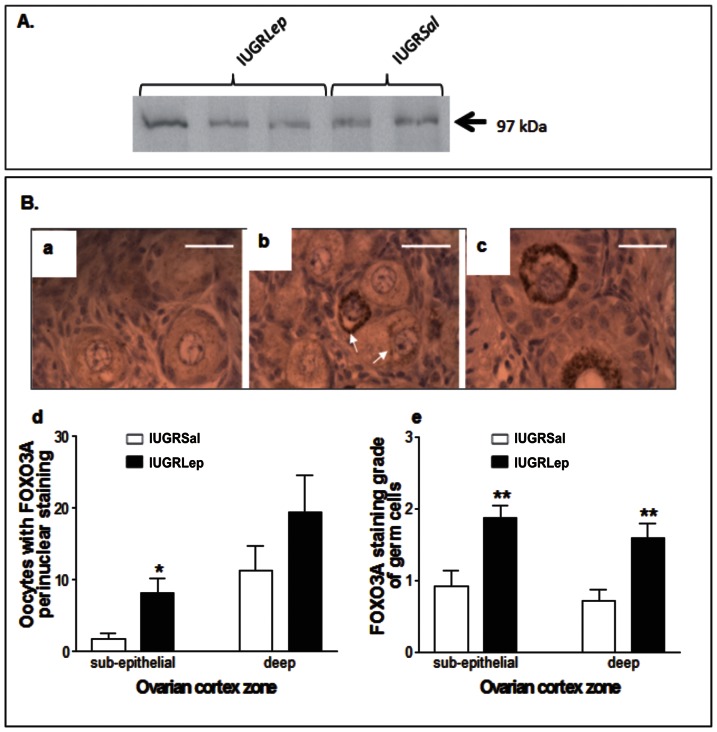
Histological structure and enzymatic activity of the pancreas of IUGR*Sal* and IUGR*Lep* piglets at d21. (A) Microscopic analysis and histological measurements in pancreas sampled from IUGR animals treated either with saline or leptin. Arrowheads highlight cells undergoing apoptosis. Bars = 10 µm. (B) Total protein content and enzymatic activities in pancreas sampled from IUGR animals treated either with saline or leptin. BW: Body weight, IU: International Unit**.** Values represent the mean ± SEM, (n = 6 per group).*: p<0.05; **: p<0.01, for leptin effect in IUGR piglet.

The overall architecture of the parenchyma of the pancreas appeared similar in all collected samples from the IUGR*Lep* and IUGR*Sal* groups. There were no changes in the numbers of cells composing the acini or in their size. As shown in Figure 4A, several isolated cells scattered throughout the exocrine parenchyma presented a cytological appearance typical of apoptosis, with a hypereosinophilic cytoplasm and a hyperbasophilic nucleus that was either shrunken (pyknosis) or fragmented (karyorrhexis). The number of apoptotic cells was significantly higher (+41.2%, p<0.05) in the leptin-treated piglets versus saline-treated piglets. There were no significant between-group differences in the numbers of cells undergoing mitosis in the pancreas parenchyma.

Biochemical analysis of pancreatic tissues revealed no differences in the total protein content between the IUGR*Lep* and IUGR*Sal* groups (Figure 4B). Analysis of pancreatic enzyme activities showed that α-amylase activity was similar in both groups, but trypsin activity was significantly increased in the IUGR*Lep* animals (+40.3%, p<0.01).

In the liver, the trabecular organization of hepatocytes and the appearance of these cells were similar in both groups. Estimates of cell proliferation based on counts of hepatocytic mitosis did not reveal any differences between the two groups (data not shown).

**Figure 5:**
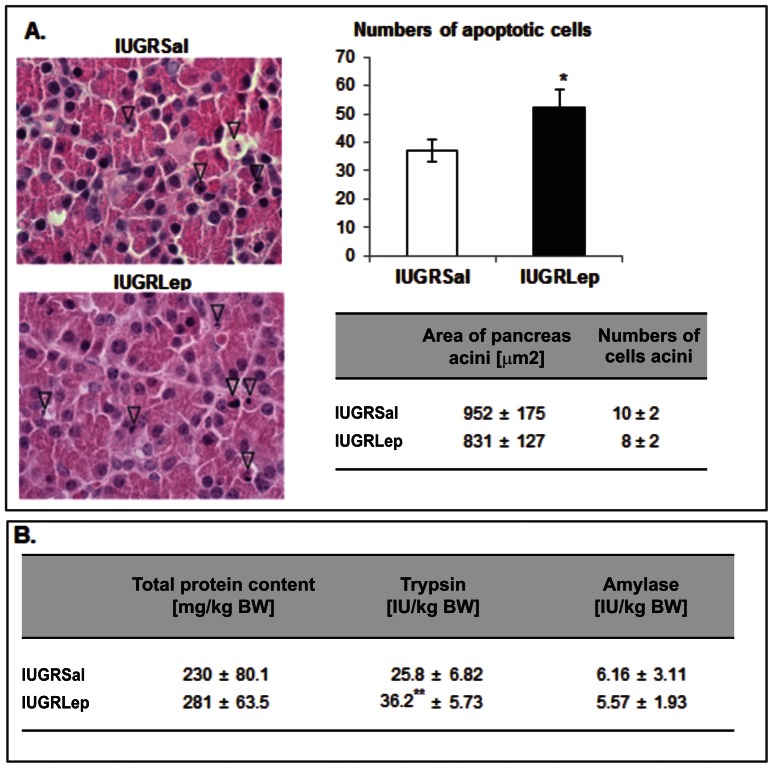
Analysis of the histological structure of secondary immune structures in IUGR*Sal* and IUGR*Lep* piglets at d21. (A) Histological structure of spleen and immunodetection of CD79+ cells in IUGR*Sal* and IUGR*Lep* piglets at d21. Left panels: Microscopic observation of white pulpe (W) in spleen samples processed with a hemalun-Eosin-Safran staining. Bars = 50 µm. Central arteriolae are indicated by arrows. Primary follicles were easily identified in IUGR*Lep* (b) and delineated with the small circle but were not visible in IUGR*Sal* (a). The inset in (b) illustrates cells undergoing intense mitotic activity (arrowhead) found in this area. Right panels: Immunolabelling for CD79 in the white pulp of IUGR piglets treated either with saline (c) or leptin (d) postnatally. (B) Histomorphometrical analysis of Peyer’s patches in IUGR*Sal* and IUGR*Lep* piglets at d21. The areas of the whole Peyer’s Patches (PP) and the B follicle were measured and the ratio corresponding to the B follicle area reported to the whole Peyer’s patche surface was determined. Values represent the mean ± SEM (n = 6 per group), *: p<0.05; **: p<0.01, for leptin effect in IUGR piglets.

In spleen samples stained with hematoxylin-eosin-safranine, primary follicles were not visible within the white pulp in the IUGR*Sal* samples, but were easily observed in the IUGR*Lep* samples (Figure 5A a–b). Interestingly, the cells were undergoing intense mitotic activity within the primary follicles of IUGR piglets treated with leptin. Immunolabeling of spleen samples using antibodies directed against the B cell surface marker CD79 highlighted the presence of aggregates of B lymphocytes in the white pulp of animals in both groups (Figure 5A c–d). The sizes of the B lymphocyte follicles were drastically reduced in the IUGR*Sal* samples as compared to IUGR*Lep* samples, and a non-negligible proportion of the B lymphocytes did not express the CD79 marker in the IUGR*Sal* samples. By contrast, IUGR piglets treated with leptin harbored numerous CD79^+^ cells that were strongly labeled in well-differentiated follicular structures.

Measurements of the surface areas of the jejunal Peyer’s patches did not indicate an effect of leptin treatment (Figure 5B), but the ratio between the follicle area normalized to the total surface of the Peyer’s patches was significantly increased (+10%, p<0.05) in the IUGR*Lep* piglets versus IUGR*Sal* animals.

While the relative weight of the kidneys was greatly increased following leptin treatment (+20%, p<0.05, Figure 1A), microscopic analyses revealed no between-group differences in either the total numbers of glomeruli (∼25 glomerules/mm^2^) or the mean size of the glomeruli (IUGR*Sal*: 2855.7±78.3 µm^2^, IUGR*Lep*: 2961.1±175.0 µm^2^).

## Discussion

Since the number of infants with IUGR is growing, it is important to understand the problems that this population may experience during growth and development [1], [29]. Most IUGR complications have been described as consequences of a lack of maturity and development of organs such as the small intestine, pancreas, spleen, kidneys, and gonads, leading to immediate defects in key biological functions [4], [30], [31], [32], [33]. In accordance with other studies in rodents highlighting the developmental role of leptin during the perinatal period in the central hypothalamus [18], [34], we previously demonstrated that leptin was also necessary for the development of several peripheral organs in the newborn rat [19]. Our previous work on the IUGR piglet showed that neonatal leptin supplementation corrected the developmental delay and adjusted the growth of several organs such as the pancreas, liver and lung to levels similar to those observed in normal birth BW animals [13]. Moreover, leptin had pronounced effects on body composition evidenced by a normalization of the BW and size as well as lean mass in the leptin treated piglets. No effects on metabolic parameters at weaning were evidenced in our both studies (glucose, cholesterol and triglycerides). One of the consequences of leptin stimulation may be an increase in growth hormone secretion. Studies performed *in vivo* in rat and pig have indeed clearly demonstrated this stimulatory effect [35], [36].

As the leptin receptor is widely expressed from an early stage of development [37], we expected that leptin acts as a key regulatory developmental factor at the cellular and molecular levels. In the current investigation we have demonstrated that leptin modulates several physiological processes involved in the development and maturation of the reproductive, immune, and gastrointestinal systems, and that leptin supplementation exerts beneficial effects in IUGR neonates.

In higher mammals, the early postnatal period represents a critical temporal window during which the termination of developmental processes occurs in many organs, correlated with the progressive acquisition of their full functionality [38], [39]. In the gastrointestinal tract, the levels and activities of the various pancreatic hydrolases and enzymes of the brush border normally increase during the postnatal period, along with growth and cellular remodeling of the small intestine and pancreas [2], [33], [40]. In the immune system of the pig, the first two weeks of postnatal life correspond to a period during which lymphatic organs such as the spleen and Peyer’s patches undergo a rapid progression in size associated with structural changes in the cellular composition and organization of lymphocyte compartments [41]. The ovary is an interesting exception to this rule, since pig ovaries are quite immature at birth, and folliculogenesis occurs postnatally [42], whereas antral follicles form and develop during the third trimester of pregnancy in human ovaries [43].

### Postnatal Leptin Treatment Enhances Ovarian Maturity in IUGR Piglets

It was previously reported that the number of primary follicles was reduced and that secondary follicles were absent in the ovaries of pigs born with IUGR compared with normally grown littermate piglets, likely due to a delay in the activation of primordial follicles [42]. In agreement with previous observations [42], [44], we found that the ovaries of 21-day-old piglets were quite immature; a substantial proportion of their germ cells were not yet enclosed in follicles but were grouped into germ cell nests or cysts. Here we have presented the first reported observation of leptin receptors in germ cells during oogenesis, before and after follicle formation, and a stimulating effect of leptin on germ cells and follicle populations in piglet ovaries.

In various mammalian species, leptin receptors are expressed in follicular oocytes from primordial stages onward [45], [46]. Moreover, leptin is known to enhance oocyte maturation i*n vitro* [47], [48] and to promote angiogenesis in ovaries, thus increasing oocyte quality [49]. Our results may indicate that leptin directly stimulated the oogonia and oocytes of piglet ovaries since (1) these germ cells were found to express leptin receptors, (2) administration of leptin induced a shift in the germ cell populations from immature stages (oogonia and oocytes in meiotic prophase) toward populations of oocytes in the dictyate stage, and (3) leptin administration enhanced the proportion of primary follicles, suggesting that leptin activated follicular growth entry. Among the leptin receptors, the full-length Ob-Rb isoform was initially considered as the functional receptor, acting through JAKs (Janus kinases) and STATs (signal transducers and activators of transcription), but now short isoforms are also known to exert important intracellular effects [50]. The presence of six different isoforms of the leptin receptors in the germ cells of the piglet ovaries suggests that various intracellular pathways may be activated by leptin. Among the most important pathways, MAP kinases have been shown to be activated by either OB-Ra or OB-Rb although to a lesser extent by the former [50], and interestingly these pathways play a key role in oocyte growth and maturation [51], [52]. Moreover, it was recently established that the PI3K/AKT pathway is the major signaling pathway of the leptin receptors in various cell types [53], [54], [55], [56], and PI3K/AKT signaling in the oocyte is known to control the survival, loss, and activation of primordial follicles in genetically modified mouse models [57]. Members of the FOXO family are downstream effectors of the PI3K/AKT pathway, and in the ovary, FOXO3A suppresses follicular activation at the earliest stages of follicular growth [58], [59], [60], [61]. In the oocytes of primordial follicles, PI3K/AKT-induced phosphorylation of FOXO3A is accompanied by its nuclear exportation, which triggers follicular activation; phosphorylated FOXO3A is later degraded in the cytoplasm [59]. We detected FOXO3A in the cytoplasm of some germ cells in piglet ovaries, in agreement with previous observations [61]. Interestingly, leptin treatment enhanced both the total numbers of stained germ cells and the numbers of oocytes with strong perinuclear staining, indicating that leptin acted upon FOXO3A protein expression in germ cells. We have tried to use different phospho-specific FOXO3A antibodies to show export from nuclei upon phosphorylation. However, we observed no staining with antibodies raised against phospho-FOXO3A (Ser253) and phospho-FOXO3A (Ser218/221), but only a faint labeling in the cytoplasm of germ cells using an antibody raised against phospho-FOXO3A (Ser294) (data not shown). Whether or not PI3K/AKT activation by leptin induces FOXO3A phosphorylation and exportation still remains to be determined. In conclusion, our observations suggest that leptin can act directly on the germ cells of piglet ovaries, thereby activating germ cell maturation and growth, but further investigations are now needed to understand its mechanisms of action on immature germ cells.

### Postnatal Leptin Treatment Enhances Digestive Function in IUGR Piglets

In pig or human IUGR neonates, a wide range of intestinal and pancreatic enzymes were previously shown to be present at lower levels than in normal birth weight subjects and were associated with reduced weight of gastrointestinal tissues [33], [62]. Chymotrypsin levels are decreased in IUGR subjects, and the activities of pancreatic lipase and trypsin are negatively correlated with the degree of IUGR [2], [63]. This scenario limits absorptive processes and nutrient utilization and induces IUGR neonate feeding intolerance, decreased fat absorption, and disposition to digestive diseases early in postnatal life, such as necrotizing enterocolitis [64].

Our observations indicated that the relative weights of the pancreas and the small intestine were increased in IUGR piglets following a six-day postnatal leptin treatment. The enzymatic activity of gut aminopeptidase N and pancreatic α-amylase were not affected by leptin treatment, but the activity of the trypsin enzyme in the pancreas of IUGR*Lep* piglets was increased, suggesting an enhanced proteolytic capacity in IUGR animals treated postnatally with exogenous leptin. Previously, the structural architecture and enzymatic activity of the brush border in the small intestines of seven-day-old normal birth BW piglets were shown to be modified under exogenous leptin treatment [65]. We did not observe such changes in our experiment for IUGR animals. This lack of morphological and functional changes between saline- and leptin-treated animals may be due to altered gut leptin sensitivity in IUGR animals. Alternatively, leptin may transiently affect the maturation of the small intestine, but this effect was not detectable in our experiments because we euthanized the animals three weeks after leptin treatment.

In the pancreas, postnatal remodeling of the parenchyma (notably in the endocrine part) is marked by an increased apoptotic activity that precipitates fetal cell destruction and allows their replacement by a new generation of cells [66]. It was previously reported that in IUGR neonates, the balance between apoptosis and proliferation is perturbed [67]. In the present study we observed that leptin treatment of IUGR piglets induced an increase in the rate of apoptosis in the exocrine parenchyma, with no changes in mitotic activity, at d21. Together with the increased enzymatic capacity of the pancreas in IUGR*Lep* piglets, these results suggest an acceleration of pancreas remodeling under postnatal leptin treatment, which merits further investigation of the effects of leptin on other pancreatic enzymes and tissue-differentiation factors. By inducing both cellular and enzymatic changes, postnatal leptin treatment ameliorates pancreas maturation in IUGR piglets and potentially provides the animals with higher proteolytic capacity. This effect may help them to better use nutrients from milk and solid starter diet during their first weeks of life, underlying the increased BW gain of *IUGRLep* piglets during the postnatal period as compared to IUGR*Sal* littermates.

### Postnatal Leptin Treatment Enhances Immune Function in IUGR Piglets

Secondary lymphoid tissues including the Peyer’s patches, lymph nodes, and the white pulp of the spleen provide an environment that enables lymphocytes to interact with each other, with accessory cells, and with antigens, resulting in the initiation of antigen-specific primary immune responses [68]. These lymphoid tissues also segregate B and T lymphocytes into specific areas. B cell lymphocytes are organized into two compartments, naive B cells and the primary follicles in which antigen-activated B cells expand and mature before becoming antibody-producing cells and memory B cells [68]. The size of the lymphocyte compartments in lymphoid tissues and their cellular compositions depend largely on age and microbial influences [69]. While these distinct B lymphocyte areas were absent in spleen and were reduced in size in the Peyer’s patches of our IUGR*Sal* animals, they were clearly developed and visible in IUGR animals treated postnatally with leptin. Human low birth BW babies with severe IUGR are prone to develop bacterial infections due to deficiencies in both the humoral and cellular immune host defenses [70]. The increase of clear B lymphocyte areas in our IUGR*Lep* group may improve the immune response to infections. Based on *in vitro* experiments and by the immune alterations observed in leptin- and leptin receptor-deficient adults animals, the role of leptin in the modulation of the immune response was evidenced [71]. Leptin controls the functions of T lymphocytes, B lymphocytes, monocytes, macrophages, and natural killer cells [71]. In the rat neonate, we previously showed that the impairment of leptin signaling altered the development of the thymus, a primary lymphoid organ [19]. In the present study, our results suggest that leptin action may exert larger effects on the immune system by also acting on secondary lymphoid structures.

### Conclusion and Clinical Perspectives

Beneficial effects of leptin remain to be assessed on organ morphology of adult pigs to determine whether there was a permanent difference or just a shift in the timing of organ maturation. Continuing our previous studies on the effects of leptin on the developmental origins of health and disease, here we have demonstrated that a six-day supply of leptin administered immediately after birth enhances the general growth of the IUGR neonate and improves the maturation of several organs, attesting to the important role of leptin in developmental processes. We observed no apparent adverse side effects of treatment at the level of the analyzed organs, suggesting that leptin administration is safe, at least in large mammals with IUGR. Further studies and large-scale investigations in pigs are planned to better define the role of leptin in general development. These studies may enable the use of leptin treatment in clinical research to reduce the problems associated with pathophysiological disorders in the IUGR animal. Finally, financial implications and the cost-efficiency of treatment of an ever-increasing list of indications should be taken into consideration before using leptin to promote organ maturation in IUGR babies.
