# Correction: Postnatal Leptin Promotes Organ Maturation and Development in IUGR Piglets

**DOI:** 10.1371/annotation/14e40c23-6fa2-4892-b843-419eb47e4db4

**Published:** 2013-09-20

**Authors:** Linda Attig, Daphné Brisard, Thibaut Larcher, Michal Mickiewicz, Paul Guilloteau, Samir Boukthir, Claude-Narcisse Niamba, Arieh Gertler, Jean Djiane, Danielle Monniaux, Latifa Abdennebi-Najar

The titles and legends for Figures 2 through 5 were incorrectly switched. 

The title and legend currently appearing with Figure 2 belong with Figure 3, the title and legend currently appearing with Figure 3 belong with Figure 4, the title and legend currently appearing with Figure 4 belong with Figure 5, and the title and legend currently appearing with Figure 5 belong with Figure 2. The Figures themselves are in correct order. 

